# Biodistribution, biocompatibility and targeted accumulation of magnetic nanoporous silica nanoparticles as drug carrier in orthopedics

**DOI:** 10.1186/s12951-020-0578-8

**Published:** 2020-01-15

**Authors:** Hilke Catherina Janßen, Nina Angrisani, Stefan Kalies, Florian Hansmann, Manfred Kietzmann, Dawid Peter Warwas, Peter Behrens, Janin Reifenrath

**Affiliations:** 1 Clinic for Orthopedic Surgery, NIFE–Lower Saxony Centre for Biomedical Engineering, Implant Research and Development, Hannover Medical School, Stadtfelddamm 34, 30625 Hannover, Germany; 2 Institute of Quantum Optics, NIFE–Lower Saxony Centre for Biomedical Engineering, Implant Research and Development, Leibniz University Hannover, Stadtfelddamm 34, 30625 Hannover, Germany; 3 Department of Pathology, University of Veterinary Medicine Hanover Foundation, Buenteweg 17, 30559 Hannover, Germany; 4 Institute of Pharmacology, Toxicology and Pharmacy, University of Veterinary Medicine Hanover Foundation, Buenteweg 17, 30559 Hannover, Germany; 5 Institute for Inorganic Chemistry, Leibniz University Hannover, Callinstraße 9, 30167 Hannover, Germany

**Keywords:** Magnetizable implant, Drug targeting, Magnetic nanoporous silica nanoparticles, PEG, Organ accumulation, Ferritic steel, Mouse model, In vivo

## Abstract

**Background:**

In orthopedics, the treatment of implant-associated infections represents a high challenge. Especially, potent antibacterial effects at implant surfaces can only be achieved by the use of high doses of antibiotics, and still often fail. Drug-loaded magnetic nanoparticles are very promising for local selective therapy, enabling lower systemic antibiotic doses and reducing adverse side effects. The idea of the following study was the local accumulation of such nanoparticles by an externally applied magnetic field combined with a magnetizable implant. The examination of the biodistribution of the nanoparticles, their effective accumulation at the implant and possible adverse side effects were the focus. In a BALB/c mouse model (n = 50) ferritic steel 1.4521 and Ti90Al6V4 (control) implants were inserted subcutaneously at the hindlimbs. Afterwards, magnetic nanoporous silica nanoparticles (MNPSNPs), modified with rhodamine B isothiocyanate and polyethylene glycol-silane (PEG), were administered intravenously. Directly/1/7/21/42 day(s) after subsequent application of a magnetic field gradient produced by an electromagnet, the nanoparticle biodistribution was evaluated by smear samples, histology and multiphoton microscopy of organs. Additionally, a pathohistological examination was performed. Accumulation on and around implants was evaluated by droplet samples and histology.

**Results:**

Clinical and histological examinations showed no MNPSNP-associated changes in mice at all investigated time points. Although PEGylated, MNPSNPs were mainly trapped in lung, liver, and spleen. Over time, they showed two distributional patterns: early significant drops in blood, lung, and kidney and slow decreases in liver and spleen. The accumulation of MNPSNPs on the magnetizable implant and in its area was very low with no significant differences towards the control.

**Conclusion:**

Despite massive nanoparticle capture by the mononuclear phagocyte system, no significant pathomorphological alterations were found in affected organs. This shows good biocompatibility of MNPSNPs after intravenous administration. The organ uptake led to insufficient availability of MNPSNPs in the implant region. For that reason, among others, the nanoparticles did not achieve targeted accumulation in the desired way, manifesting future research need. However, with different conditions and dimensions in humans and further modifications of the nanoparticles, this principle should enable reaching magnetizable implant surfaces at any time in any body region for a therapeutic reason.

## Background

Implant-associated infections represent one dreaded complication in orthopedics. They occur as a result of contamination during or immediately after surgery or at later periods following hematogenic spread [1–3]. Numbers of implantations, in general, are growing because of an aging population, increasing obesity, and other predisposing factors [4–7]. In addition, worldwide rising bacterial resistance against antibiotics [8–11] and further, the irreversible adhesion of bacteria and production of extracellular matrix in biofilm formation on the implant surface (infection cause) complicate a successful treatment [12–14]. This challenge is still accomplished by using high systemic doses of antibiotics for several months, accepting adverse side effects [1, 15, 16]. Apart from intensive costs, this leads to high burdens for the patient as well as risks like amputation of the affected limb or even death in case of treatment failure [5, 17]. So far, different prevention methods were studied avoiding bacterial adhesion in combination with enhancing osseointegration [18, 19] like surface modifications or antimicrobial coatings of implants [20–23].

As a treatment strategy in case of occurring implant-associated infection in orthopedics, implant-directed magnetic drug targeting (ID-MDT) represents a possible approach to reduce systemic antibiotic doses, period and therefore side effects and probably the need of revision surgeries. Locally high and sufficient levels of antibiotics might be achieved by loading magnetic nanoparticles with these antibiotics and target them by magnetic force towards the implant, followed by triggered drug-release [24–26].

In the last years there has been certain skepticism as to whether the various biomolecular targeting mechanisms (“vectorization” using certain receptors on target cells, antibody-antigen interactions, etc. [27, 28]) are actually effective and ultimately transferable to the human organism [29]. Accordingly, the question “Does nanomedicine have a delivery problem?” is discussed intensively [30]. Therefore, the necessity to develop new approaches is still present.

Magnetic nanoparticles are already versatilely used in research and partly in clinical issues for hyperthermia or drug delivery in tumor [31–35] and infection treatment [36, 37], as contrast agents for magnetic resonance imaging [38–40], and others [41, 42]. The biocompatibility of certain magnetic nanoparticles with different composition, magnetic properties or size has already been published [43, 44]. Surface modifications with polyvinyl alcohol, polyethylene glycol (PEG, used in this study) or dextran, among others, can be performed to protect particles from rapid capture out of the bloodstream by the immune system, particularly by the mononuclear phagocyte system (MPS) [45–48]. Nevertheless, undesired particle uptake into different organs occurs, for example into the lung, liver, and spleen following intravenous administration [43, 49] and has to be minimized. To our knowledge, no studies were performed dealing with in vivo extravasation of magnetic nanoparticles towards the surface of the magnetic source. However, it is presumed that an external (electro-) magnetic field alone is insufficient to attain accumulation of magnetic nanoparticles in deeper body regions [50, 51]. For that reason, the here presented study used a magnetizable plate as a representative for orthopedic implants/prostheses as a second source of a magnetic field. When magnetized by the external magnetic field it will intensify the existing field gradient [25, 52].

The design of this study is significantly different from previous publications since the applied therapy approach will enable a locally effective treatment at any time and in any body region, making the normally inaccessible implant surface reachable for therapeutics.

Our preliminary in vivo experiments demonstrated the detectability of fluorescence-labelled magnetic nanoporous silica nanoparticles (MNPSNPs) after subcutaneous administration onto inserted magnetic test and paramagnetic control implants [53]. The study presented here dealt with follow-up experiments which focused on the MNPSNP performance after intravenous application. Three central problems were examined in vivo: Firstly, the MNPSNPs were supposed to be biocompatible. This property was examined for a duration of up to 42 days. Secondly, it was hypothesized that the MNPSNPs were available in the implant area to a large extent due to PEG-surface with associated prolonged blood half-life, as well as enabled extravasation of MNPSNPs assuming comparably increased permeability as reported for similar but smaller nanoparticles in a study by Qiu et al. [54]. Thirdly, based on our preliminary results, it was assumed that ferritic steel 1.4521 implants should attract significantly higher numbers of magnetic nanoparticles than paramagnetic titanium alloy (Ti90Al6V4) implants in vivo. To verify these hypotheses, test and control implants were inserted subcutaneously followed by intravenous administration of fluorescent MNPSNPs and immediate application of an electromagnetic field in a mouse model.

## Methods

### In vivo setup

The in vivo experiments were authorized according to the German Animal Welfare Act (registration number: 33.12-42502-04-13/1103) and performed in 50 female BALB/cJHanZtm mice with an average body weight (BW) of 28 ± 2.4 g. Mouse husbandry was organized in groups of up to five mice with a 14 h/10 h-day/night cycle and free access to food (Maintenance diet, Altromin Spezialfutter GmbH & Co. KG, Germany) and tap water.

Ferromagnetic implants (n = 50, 6 × 2 × 1 mm^3^, ferritic stainless steel 1.4521, Outokumpu Nirosta GmbH, Germany) with high relative permeability and low residual magnetization (remanence) were inserted subcutaneously. Each mouse received one implant at the left hindlimb, parallel to the femur. Paramagnetic titanium alloys Ti90Al6V4 with the same dimensions (n = 50, GoodFellow, England) were similarly inserted in the contralateral hindlimb serving as a negative control.

For the surgical procedure anesthesia was performed by intraperitoneal injection of a ketamine-xylazine-mixture (70 mg ketamine/kg BW (Wirtschaftsgenossenschaft deutscher Tierärzte eG, Germany) and 7 mg xylazine/kg BW (CP-Pharma Handelsgesellschaft mbH, Germany)), occasionally prolonged by midazolam (5 mg/kg BW, i.p., ratiopharm GmbH, Germany). Peri-operative analgesia was ensured by subcutaneous administration of meloxicam (1 mg/kg BW, CP-Pharma Handelsgesellschaft mbH, Germany). The implant was inserted after skin incision and the wound was closed by two horizontal mattress sutures with PROLENE^®^ 6-0 (Johnson & Johnson Medical GmbH Ethicon Germany).

After the surgical procedure, 420 µg MNPSNPs dispersed in 0.1 mL sodium chloride were injected intravenously in the mouse’s tail vein, resulting in a mass concentration of approx. 230 µg MNPSNPs/mL blood. The used MNPSNPs have a Fe_3_O_4_-core, a 50 nm thick silica shell, an average diameter of approx. 112 ± 16 nm, a spherical shape, and superparamagnetic properties. Furthermore, the nanoparticles are negatively charged (zeta-potential: − 30 mV) and modified with rhodamine B isothiocyanate (RITC) enabling detection and polyethylene glycol-silane (PEG) prolonging half-life in blood.

As immediately following final step, a magnetic field was applied at both hindlimbs (strength approx. 1.8 T, EM2, Magnet-Messtechnik J. Ballanyi, Germany) for 10 min. The detailed surgical procedure and magnetic field application, as well as the synthesis and characterization of the MNPSNPs, have already been described by Janßen et al. [53].

To obtain an overview of MNPSNP distribution in the body, mice were euthanized by cervical dislocation at different time points after MNPSNP injection: 15 min, 1, 7, 21, 42 days (group 0, 1, 7, 21, 42), ten mice per group. For evaluation, the implants were removed, and blood, urine and organ samples were taken. During the postoperative follow-up, the mice were examined clinically every day for the first week and three times per week afterward (except for group 0).

### Biodistribution of MNPSNPs detected by fluorescence analysis and pathological changes

Blood and urine as well as organ material from lungs, liver, spleen, kidneys, and exemplarily from the brain (0.4 × 0.3 × 0.3 mm^3^, respectively) were spread out homogeneously onto slides, the so-called blood, urine, and organ smear samples. Furthermore, 5 µm thick histological slices of formaldehyde 4%-fixed and paraffin-embedded organs including muscle, subcutis, and skin of the hindlimbs (area of former implant location), exemplarily of the heart muscle, tail vein, *Vena cava caudalis* and its branches, *Lnn. iliaci*, *poplitei* and *subiliaci* were produced. Blood, urine, and organ smear samples as well as histological slices were analyzed by fluorescence microscopy (Axioskop 40, Carl Zeiss AG, Germany) using 400fold magnification, a red filter for characteristic detection (filter set 20, Excitation BP 546/12, Beam Splitter FT 560, Emission BP 575-640, Carl Zeiss AG, Germany) and a green filter (filter set 44, Excitation BP 475/40, Beam Splitter FT 500, Emission BP 530/50, Carl Zeiss AG, Germany) for the control of autofluorescence.

Ten fields of view of each blood, urine, and organ smear sample were scored regarding the presence of the irregularly shaped and different sized MNPSNP clusters (Table 1) and summed up to a total score, as previously described [53]. For the verification of these results, the presence of MNPSNP clusters in unstained histological slices was analyzed descriptively with regard to its quantity, size, shape, localization, distribution, association or pattern. Hematoxylin–eosin (H.E.) stained histological slices of all mentioned organs were descriptively evaluated for pathological changes and eventually detectable MNPSNP clusters by an unblinded investigator as well as an investigator unaware of the treatment assignment. Additionally, exemplary fresh lung, liver, spleen and kidney samples from mice in groups 0, 1, and 7 were sprinkled with 0.1% riboflavin (Sigma Aldrich) in phosphate-buffered saline for 5 min for further examination with a multiphoton microscope (MPM200, Thorlabs, Germany). A tunable femtosecond laser system (titanium-sapphire laser, Chameleon Ultra II, Coherent Inc., USA) at a wavelength of 850 nm and an objective with a numerical aperture of 1.05 (Olympus XLPLN25WMP2, Germany) were used for imaging.

**Table 1:** Score for semiquantitative evaluation of MNPSNP distribution and accumulation of blood, urine, organ smear and droplet samples according to Janßen et al. [53]

Cluster size	Quantity				
	None (0)	Occasional (1–5)	Few (≤ 20)	Many (≤ 100)	Plentiful (≥ 100)
Very small	0	1	2	3	5
Small	0	2	3	4	6
Medium	0	3	4	5	7
Large	0	4	5	6	8
Very large	0	5	6	7	9

Results were additionally compared with physiological, untreated organ samples which were received from mice that have been killed according to §4 of the German Animal Welfare Act and reported according to the legal requirements.

### Detection of targeted enrichment of MNPSNPs on the implant surface

The ferritic steel and titanium alloy explants were put into 100 µL *A.* *dest.*, respectively, vortexed and treated in an ultrasonic bath to detach eventually accumulated MNPSNPs. This procedure was repeated for another two times, always transferring the treated explant into new *A.* *dest*. The three resulting suspensions for each explant were dropped on slides, five drops per suspension, so-called droplet samples. With the same settings for fluorescence microscopy as described for the blood, urine, and organ smear samples, one visual field for peripheral regions and three visual fields of the middle region of each dried drop were scored regarding the quantity and size of MNPSNP clusters (Table 1). Finally, a total sum score of suspensions 1–3 was calculated (possible score range per suspension: 0–700; in total: 0–2100).

Afterward, the surfaces of the explants were evaluated by fluorescence microscopy with the same settings. Possibly remaining MNPSNPs were descriptively assessed regarding distribution and quantity.

More detailed descriptions of production and evaluation of organ smear and droplet samples have already been described by Janßen et al. [53].

### Statistics

The final evaluation and statistical analysis included a total of 41 animals. Nine animals dropped out for final evaluation due to terminal circulatory collapse during or after anesthesia (n = 4) or failed injection of MNPSNPs (n = 5). The following number of mice for each group was evaluated: n_0_ = 8, n_1_ = 9, n_7_ = 9, n_21_ = 6, n_42_ = 9.

Statistical analysis was performed using SPSS^®^ 25 (IBM, USA). Nonparametric tests (Kruskal–Wallis/Mann–Whitney-U) were performed in blood, organ smear, and droplet samples due to ordinal evaluation methods. If p < 0.05, differences between the time groups (blood, organ smear and droplet samples) and additionally between the implant materials (droplet samples) were considered statistically significant.

## Results

### Clinical examination of mice in the follow-up periods

The area of wound suture was mildly swollen, reddened and scabbed for the first days after surgery. No other clinical changes were observed. Furthermore, the mobility of the hindlimbs was not restricted by the implants. During the postoperative follow-up, no mouse lost temporary more than 5% of BW.

### Two distributional patterns of MNPSNPs in organs were detected via fluorescence microscopy

**Fig. 1:**
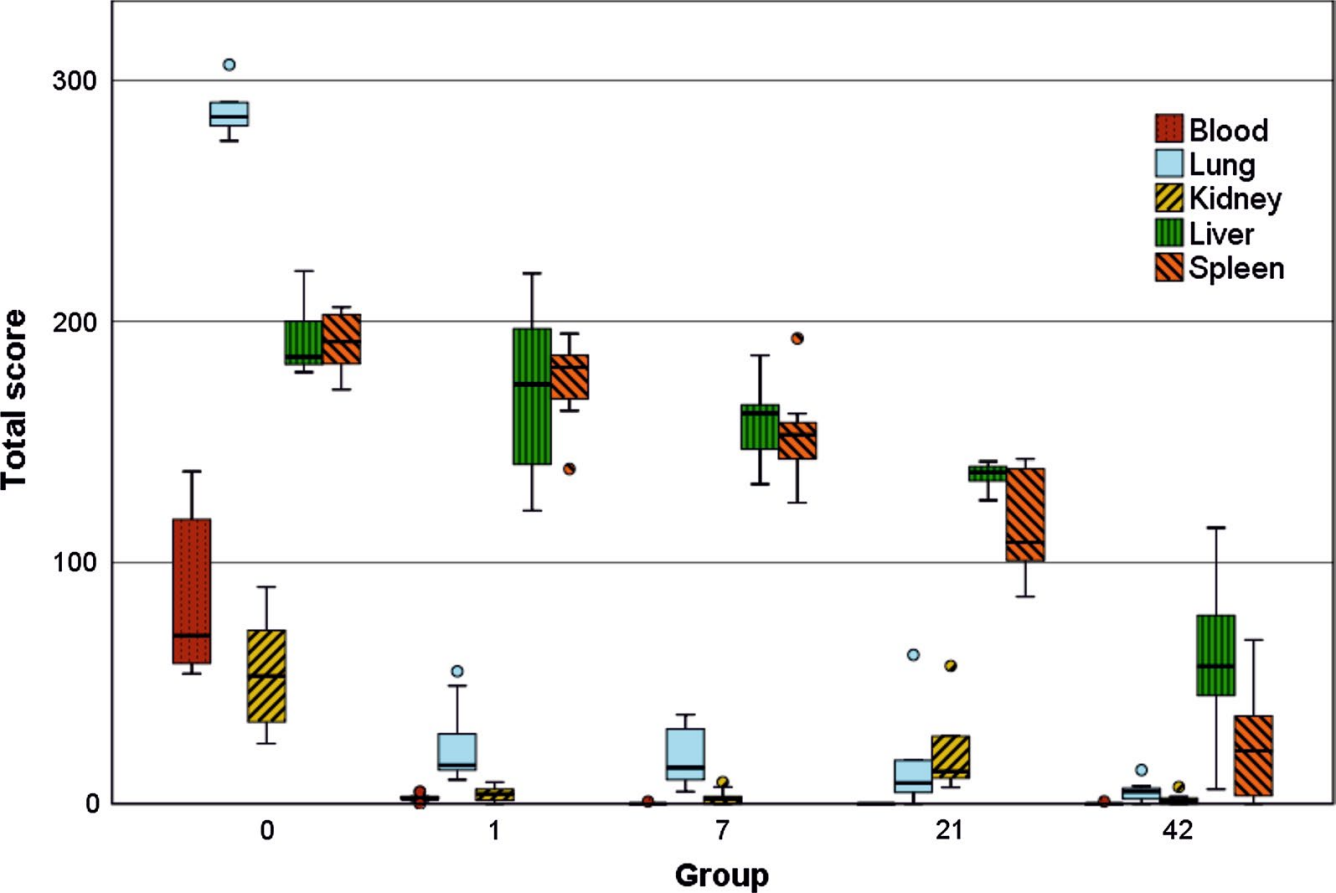
Box-and-whisker plots of the summed score regarding quantity of MNPSNP clusters in blood and organ smear samples per group (0, 1, 7, 21 and 42). The boxes represent the 25th to 75th percentiles, the black solid lines indicate the median values and circles show outliers. For statistical significances, see Table 2

All applied methods, including smear samples, fluorescence microscopy of histological slices and multiphoton microscopy (MPM) of exemplary organs, showed congruent results. The summed scores of blood and smear samples are shown in Fig. 1 and its significances are listed in Table 2.

**Table 2:** Overview of statistical significances (exact *p* value) corresponding to Fig. 1 (blood and organ smear samples) and Fig. 7 (droplet samples) created by Mann–Whitney-U-Test

Compared groups	Smear samples					Droplet samples	
	Blood	Lung	Kidney	Liver	Spleen	Ferritic steel	Titanium alloy
0–1	***	***	***		*	**	***
0–7	***	***	***	**	**		
0–21	**	**	*	**	**		
0–42	***	***	***	***	***	*	*
1–7	**				*	**	*
1–21	**		**	*	**	***	***
1–42	**	***		***	***	***	***
7–21			**	*	**	***	**
7–42		**		***	***	***	***
21–42			**	***	***		

Groups 0, 1, 7, 21 and 42, *p < 0.05, **p < 0.01, ***p < 0.001

**Fig. 2:**
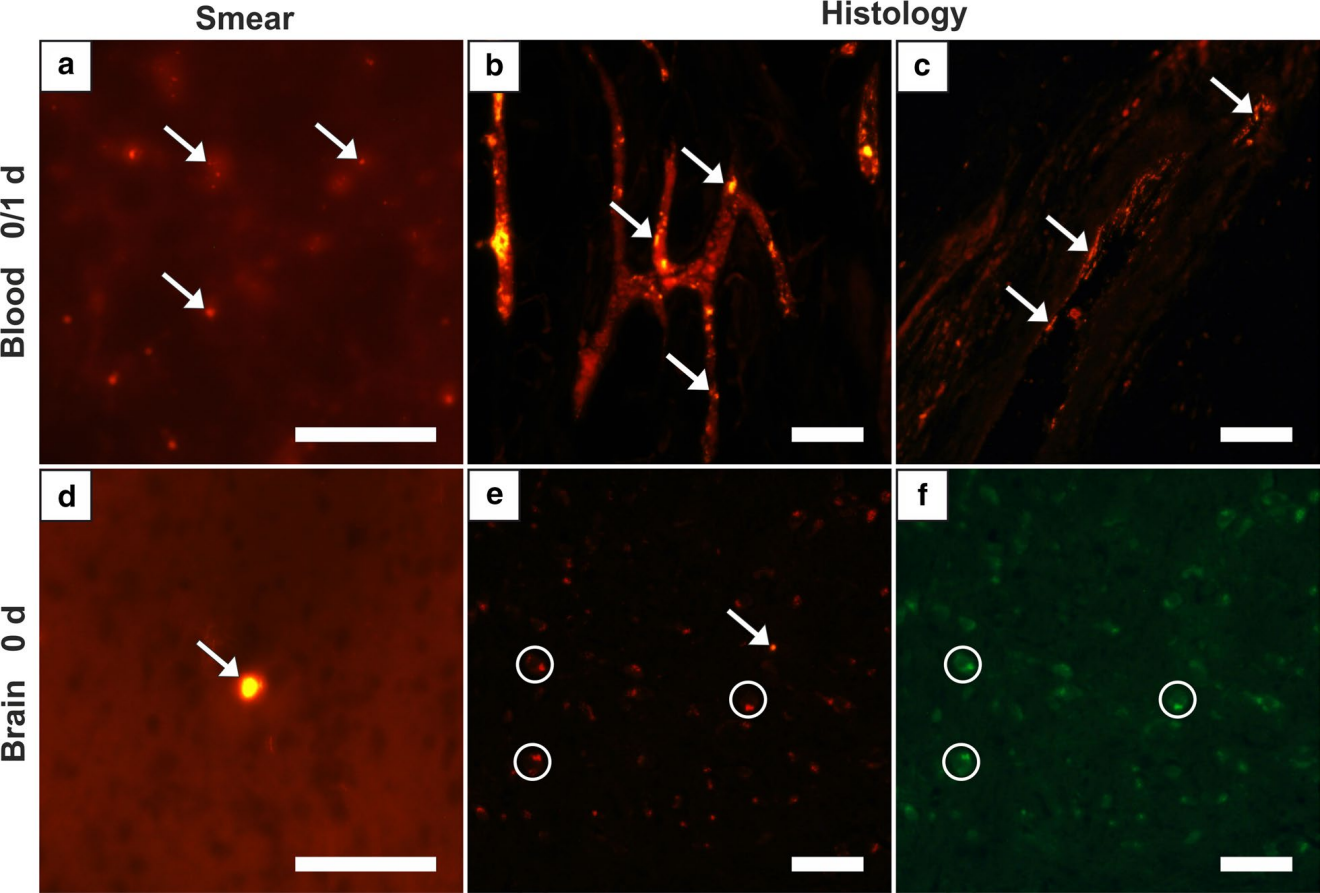
Detection of MNPSNP clusters (→) via fluorescence microscopy in blood (**a**–**c**) and brain (**d**–**f**) smear samples (**a**, **d**) and histological slices (**b**, **c**, **e**, **f**). **a** Blood with many MNPSNP clusters, group 0; **b** small blood vessels in a tail with large amounts, group 0; **c** blood vessel with clusters lining its wall, group 1; **d** brain with one large cluster, group 0; **e** brain with a single cluster, group 0; **f** see **e**, green filter for control and demonstrating autofluorescent cells (circles). All scale bars: 50 µm

The evaluation of MNPSNP quantity in blood and different organs revealed two distributional patterns: (1) a high MNPSNP concentration immediately after intravenous injection followed by a significant drop and (2) a high concentration followed by only slow decrease. The first pattern was observed in blood, heart muscles, lungs, kidneys, and brains. The highest MNPSNP concentration existed in group 0, respectively, followed by a highly significant decrease towards zero until the next day. The blood samples, as well as histological slices of the tail vessels, *Vena cava caudalis* and its branches, contained large amounts of MNPSNP clusters (Fig. 2a–c). In group 1 some vessels showed clusters wide-stretched in the area of vascular walls as if they were coating these. One very small caliber vessel was filled with clusters even in group 7. Exemplarily sliced hearts showed occasional, diffuse and different-size MNPSNP clusters in the muscle up to one day.

Partly strong autofluorescence of various cells massively impeded MNPSNP detection in the brain. Solely in group 0 single, rare and little to large clusters could be identified in histological brain samples and also in exemplarily taken brain smear samples (Fig. 2d–f).

**Fig. 3:**
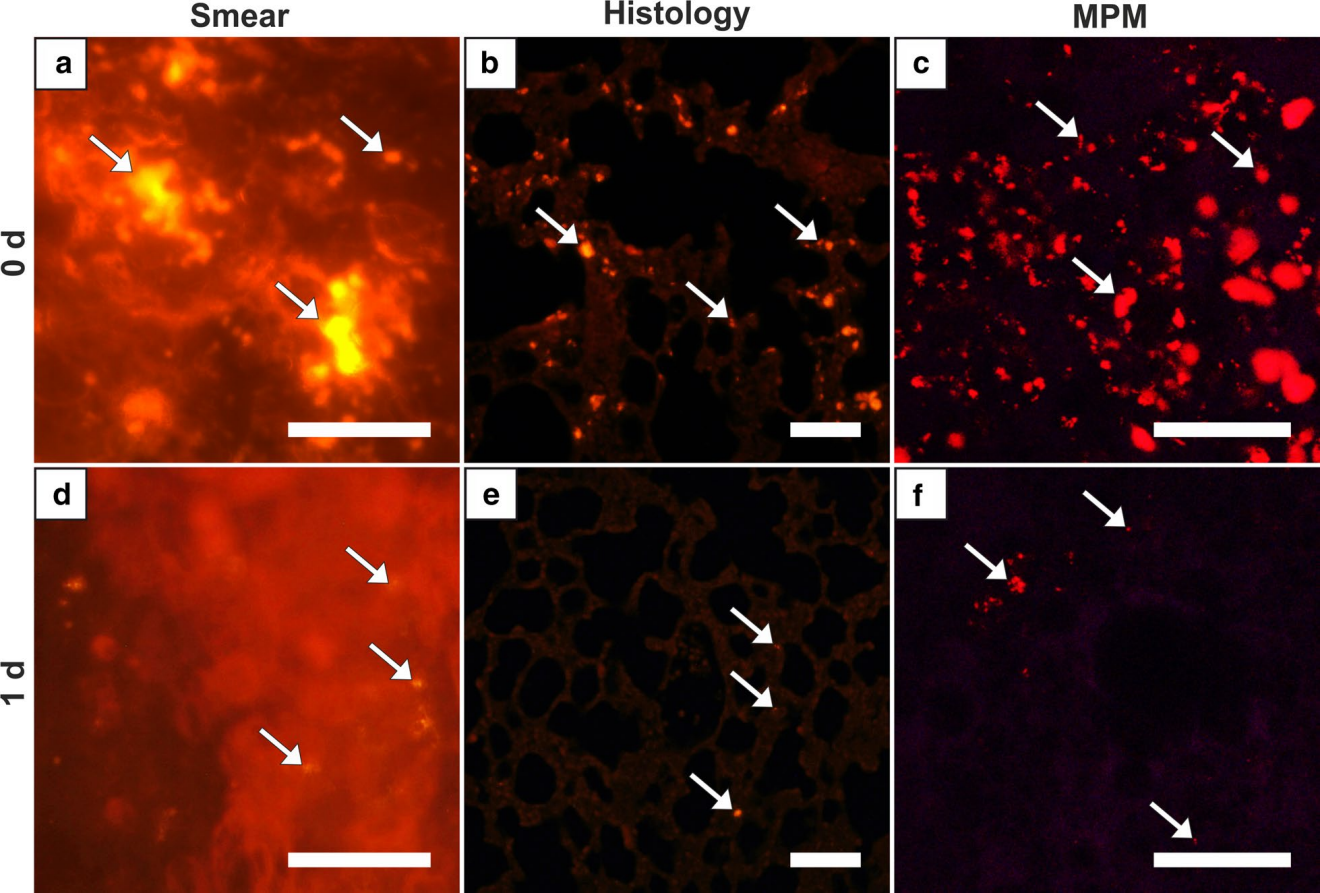
Lung. Detection of MNPSNP clusters (→) in smear samples (**a**, **d**), histological slices (**b**, **e**) via fluorescence microscopy and fresh tissue via multiphoton microscopy (MPM; **c**, **f**). **a**–**c** Group 0 with moderate to high amounts of MNPSNP clusters of different size and shape in alveolar septa **b**; **d**–**f** group 1 with sporadic to rare clusters. All scale bars: 50 µm

The lung of group 0 showed by far the highest score of all organ samples (Fig. 1, Table 2). Histologically, partly high-grade, diffuse MNPSNP-characteristic fluorescence of different sizes and shape were found in alveolar septa (Fig. 3b). In total, the entire lung was affected moderately to severely. In group 1 MNPSNPs became sporadic and rare (Fig. 3d–f). Sliced corresponding lymph nodes in groups 0 and 1 very rarely contained MNPSNP clusters.

**Fig. 4:**
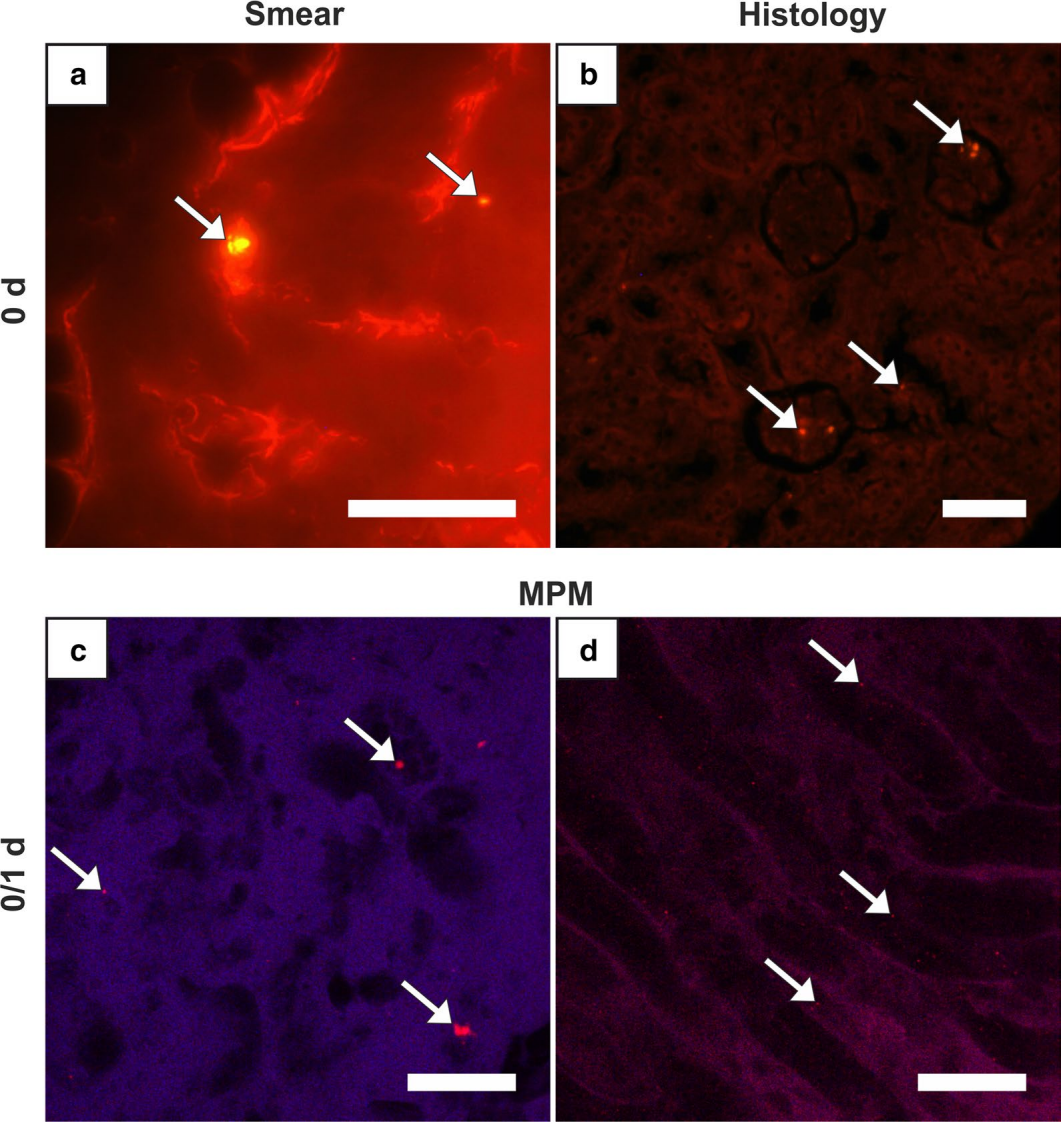
Kidney. Fluorescence detection of MNPSNP clusters (→) of group 0 (**a**–**c**) and group 1 (**d**). **a** Smear sample with a single large cluster; **b** histological slice with single large clusters in some glomeruli; **c**, **d** Images of MPM with clusters in the tubule lumens (**c**) and finely distributed in tubule epithelium (**d**). All scale bars: 50 µm

There was no difference observed between right and left kidneys. In group 0, a low amount of mostly large clusters was detected in a lot of glomeruli (Fig. 4b), as well as diffuse, small clusters in the areas of tubules in medulla and cortex. At later periods, clusters were occasional to rare. Corresponding smear samples corroborated this pattern but showed a small peak in group 21 with a significant decline towards group 42 (Fig. 1, Table 2). Autofluorescence of the tissue moderately impeded the detection. In the images of MPM, clusters were detectable in the lumens and in group 1 and 7 very small clusters were finely distributed in tubule epithelium or in lumens (Fig. 4c, d). MNPSNP detection in urine samples for excretory behavior was totally impossible due to extreme autofluorescence.

**Fig. 5:**
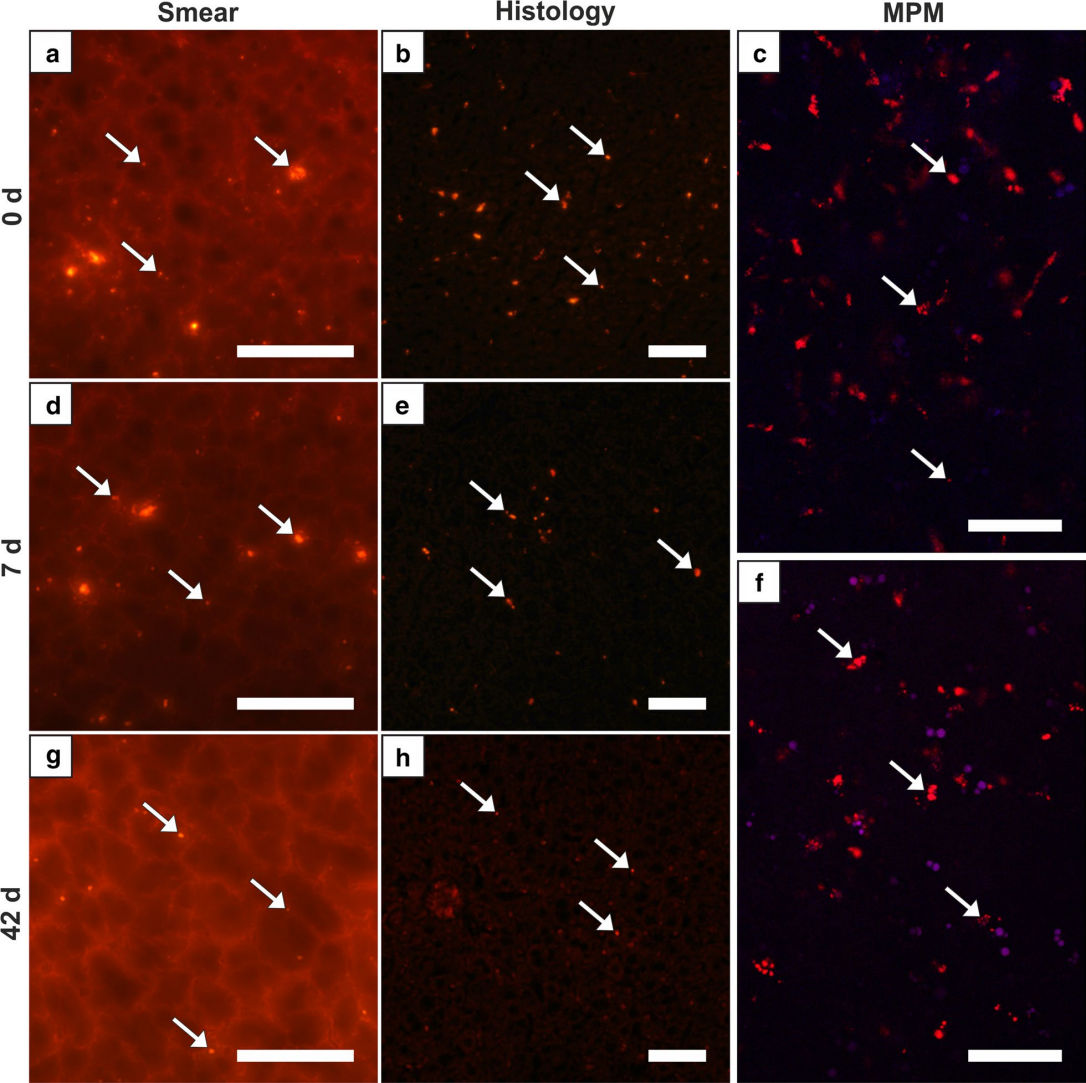
Liver. Detection of MNPSNP clusters (→) in smear samples (**a**, **d**, **g**), histological slices (**b**, **e**,** h**) via fluorescence microscopy and fresh tissue via MPM (**c**, **f**). **a**–**c** Group 0, **d**–**f** group 7, mild to moderate, diffuse, differently sized clusters, partly grouped together in oval shape, respectively; **g**, **h** group 42, occasional, diffuse clusters. All scale bars: 50 µm

**Fig. 6:**
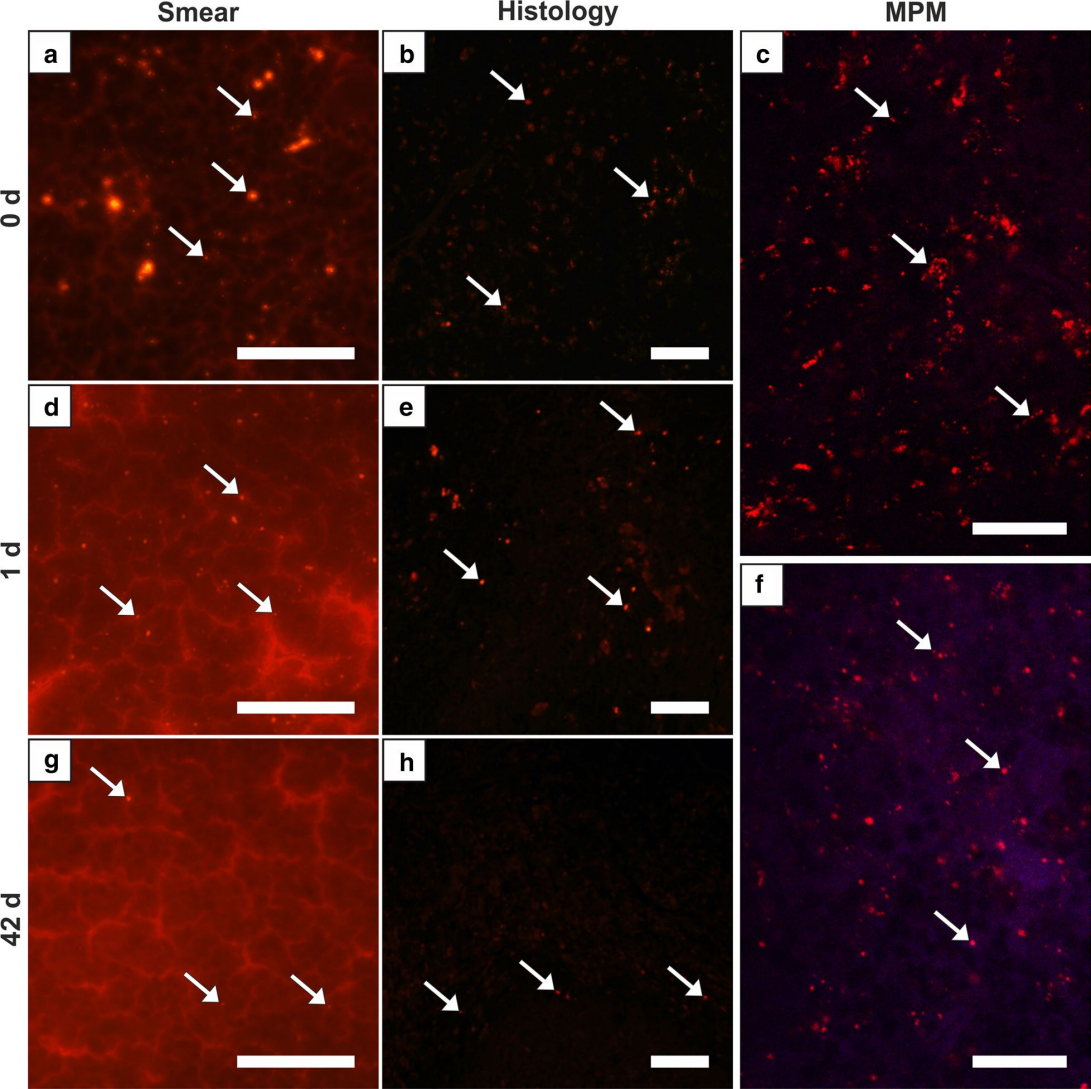
Spleen. Detection of MNPSNP clusters (→) in smear samples (**a**, **d**, **g**), histological slices (**b**, **e**, **h**) via fluorescence microscopy and fresh tissue via MPM (**c**, **f**). **a**–**c** Group 0, mild to moderate, diffuse, differently sized clusters, partly grouped together in oval shape; **d**–**f** group 1, slightly less than group 0; **g, h** Group 42, rare to occasional, diffuse clusters. All scale bars: 50 µm

The second pattern was a high MNPSNP concentration in group 0 with a slow decrease towards later time groups, observed in liver and spleen (Figs. 5, 6). Histologically, livers contained mild to moderate, diffuse, differently sized MNPSNP clusters which were not observed in core areas of hepatocytes. Finally, in group 42 they occurred occasionally. In the red splenic pulp (mostly in marginal sinuses and directly around the follicles), MNPSNP-characteristic fluorescence was low-graded, diffuse, oligofocal highly concentrated, in group 7 still mild to moderate and even in group 42 detectable. In both organs, clusters were mostly single or grouped together in oval shape, which was clarified by images of MPM. The high score values of hepatic and splenic smear samples in group 0, even if lower than lung values, just slowly decreased and as late as in group 42 significantly declined (Fig. 1, Table 2).

Finally, it should be noted that the exact localization of clusters—whether present in a tiny blood vessel/capillary or in the heart muscle/lung septa/brain tissue itself—could not be spotted.

### Targeted accumulation of MNPSNPs on the implant and in its surrounding tissue

**Fig. 7:**
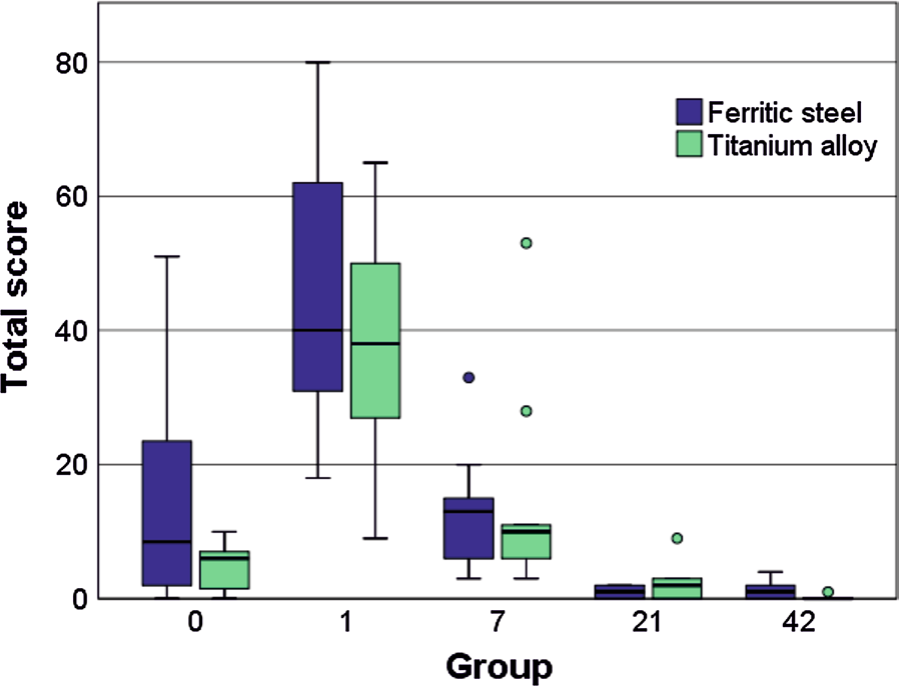
Box-and-whisker plots of the evaluation of droplet samples representing MNPSNP mass accumulated on the surface of ferritic steel and titanium alloy implants. Summed score respectively is shown for the examined groups 0, 1, 7, 21 and 42. The boxes represent the 25th to 75th percentiles, the black solid lines indicate the median values and circles show outliers. For statistical significances, see Table 2

MNPSNP clusters on implants and in the surrounding tissue were already detected directly after magnetic field application (group 0), followed by a significant increase after one day (group 1) and a decline of almost exponential character until day 42. There were no significant differences between ferritic steel and titanium alloy except for group 42 (*). The scores of droplet samples are shown in Fig. 7 and its significances are listed in Table 2.

Regarding the presence of still remaining MNPSNPs on the explants, sometimes very little clusters cannot be excluded because of impeding autofluorescence of adherent tissue/cells, especially on skin-facing sites or focal areas of the ferritic implant material itself. In group 0 and 7, only on ferritic steel explants single remaining little clusters were detected. On the contrary, in group 1 a lot of titanium and most ferritic steel explants contained occasional, diffuse, small clusters. In a piece of adherent tissue on one ferritic plate a moderate amount of MNPSNPs was found. No characteristic fluorescence was observed in group 21 or rather 42.

**Fig. 8:**
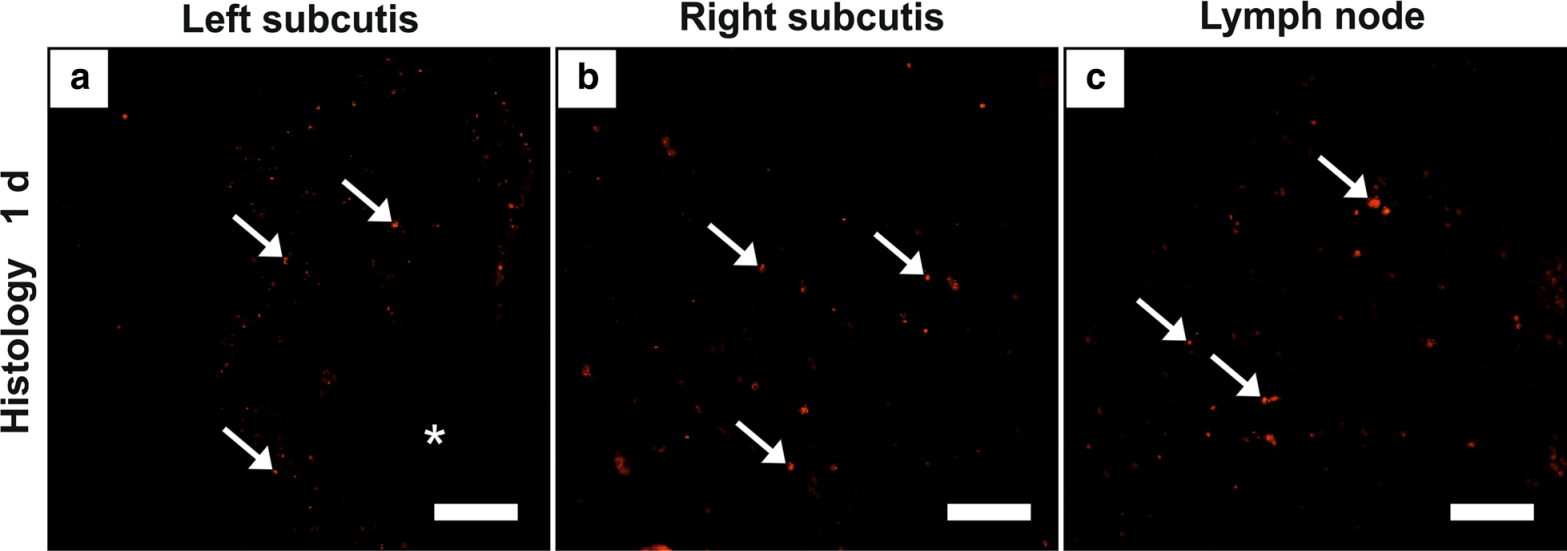
Detected MNPSNP clusters (→) in subcutis with former implant location (*) of the left (**a**) and right hindlimb (**b**) and in a corresponding lymph node (**c**) of group 1. Scale bar: 50 µm

Histological slices of subcutis where the implant had been located showed diffuse, single, up to large MNPSNP clusters in surrounding muscle, connective tissue, and subcutaneous fat tissue until 7 days via fluorescence microscopy. Thereafter, detection was improbable. If the interface was cut, marginal to moderate amounts were found distributed also in inflamed tissue and partly associated to cells in group 1. Differences between the left and right hindlimbs could not be observed (Fig. 8a, b).

In individual cases, corresponding lymph nodes (*Lnn. iliaci*, *Lnn. poplitei*) contained single little to large clusters only in group 0 and 1 (Fig. 8c). Strong autofluorescent cells impeded a definite detection of partly weak fluorescent MNPSNP clusters. Therefore, the presence of very small clusters cannot be excluded.

### No pathomorphological changes in most inner organs and confirmed MNPSNP detection via H.E. staining

**Fig. 9:**
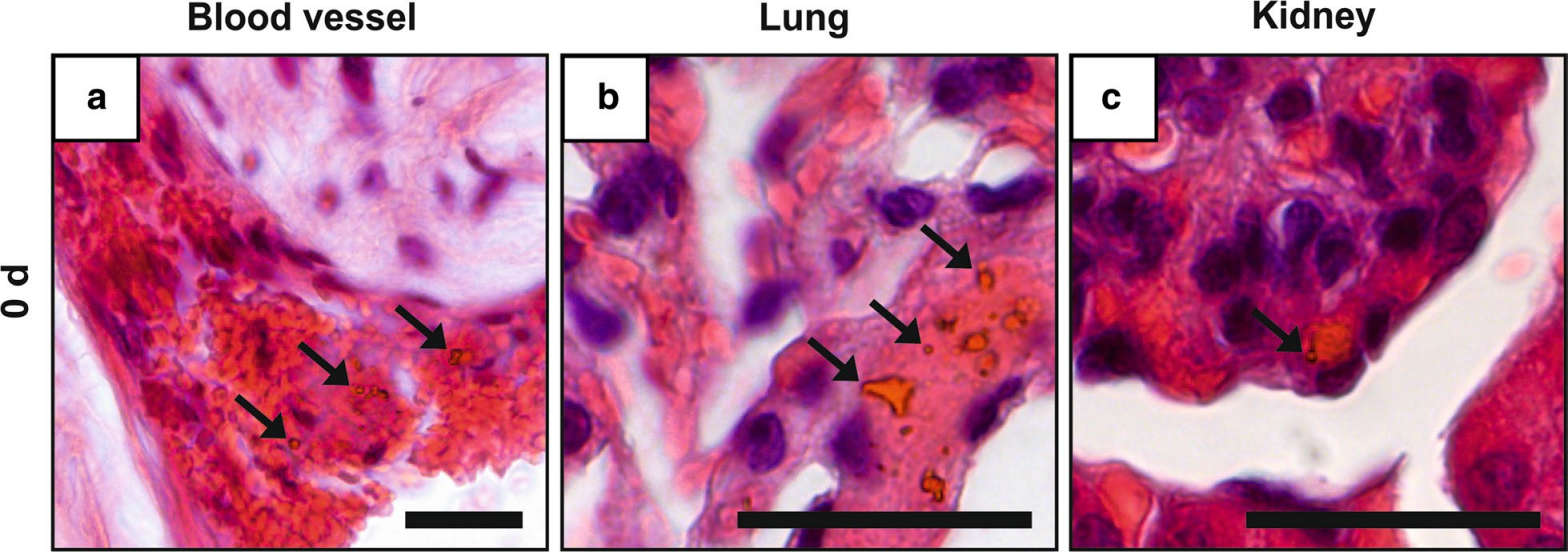
MNPSNP cluster (→) detection in H.E. stained histological slices in a blood vessel (**a**), alveolar septa (lung, **b**) and glomeruli (kidney, **c**). All scale bars: 50 µm

**Fig. 10:**
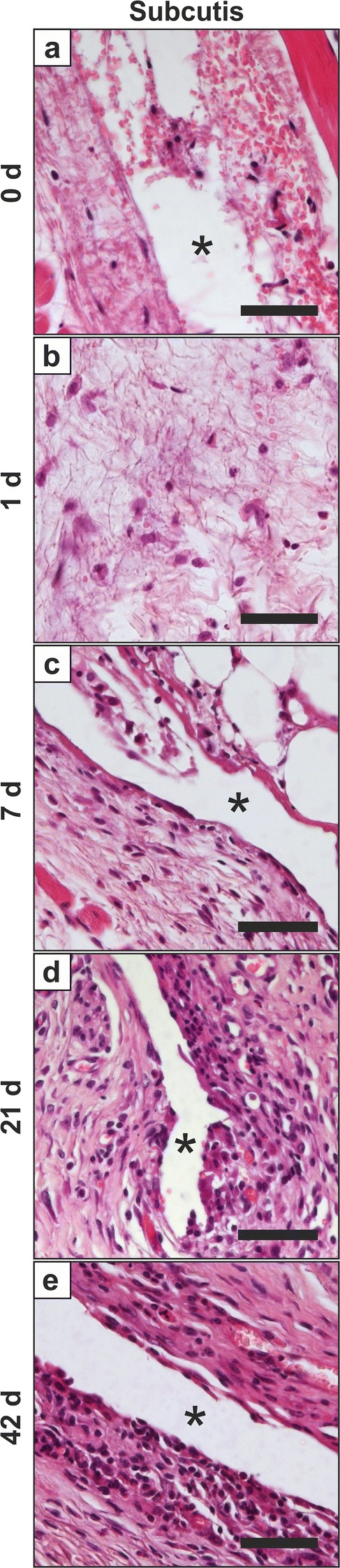
Pathological findings in the subcutis at the implantation site (*). **a** Mild focal hemorrhage with fibrin extravasation, few neutrophils and macrophages, group 0; **b** low numbers of neutrophils and macrophages with fibrin, group 1; **c** mild lympho-histiocytic inflammation, group 7; **d**, **e** mild lympho-histiocytic inflammation with fibrosis, group 21 (**d**) and 42 (**e**), respectively. H.E. staining, all scale bars: 50 µm

The results of both pathological reports were consistent. MNPSNPs were detected in H.E. stained samples as irregular shaped, homogenously brown particles. Solely in group 0 MNPSNPs were occasionally found in tail vessels, *Vena cava caudalis* and its branches, as well as rarely in the glomeruli of kidneys. In the lungs, many MNPSNP clusters were detected in alveolar septa in group 0 (Fig. 9) but also some clusters in group 1. Apart from the detection of MNPSNPs, no significant pathomorphological alterations were found in the kidneys, spleens, brains, lymph nodes, and heart muscles. In some animals a mild, multifocal, lymphohistiocytic inflammation in lung and/or liver was detected at all time points. Similar alterations in the subcutis at both implantation sites (right and left hindlimbs) were detected ranging from acute inflammatory changes consisting of fibrin intermingled with few neutrophils and macrophages in group 0 and 1 to mild lymphohistiocytic inflammation and fibrosis in the latest groups (Fig. 10).

## Discussion

In the present study, three main hypotheses were examined. Firstly, it was assumed that systemically administered MNPSNPs are physiologically harmless to the body. Secondly, it was hypothesized that MNPSNPs are available in the implantation area. And thirdly, based on the results of previous in vitro and in vivo studies [26, 53, 54], an externally magnetized ferromagnetic implant material was supposed to be able to accumulate these nanoparticles at the implant surface in higher concentrations than the control. This would mean a safe use of MNPSNPs as future drug carrier system for implant-associated infection treatment.

According to the first hypothesis, we can state that MNPSNPs are biocompatible and do not seem to influence the body’s physiology, at least in the observed time interval of up to 42 days. No clinical changes of mice and no significant pathomorphological alterations in histological examination were observed which coincides with different nanoparticles in the literature [43, 44, 55]. The mild, multifocal inflammatory alterations in lungs and/or livers were supposed to be not MNPSNP-associated since they occurred in all groups infrequently and lesions were not associated with MNPSNP clusters.

Regarding the second hypothesis, the significant decline of MNPSNPs in group 0 in blood samples and high amounts in examined organs, especially the lung, demonstrate a fast capture. Concerning the biodistribution of MNPSNPs after intravenous injection, the lung with its very small capillaries and phagocytose system represents the first bottleneck for nanoparticles and an undesired direct entrapment probably by different lung macrophages or monocytes [49, 56, 57]. The detection of MNPSNP clusters in the lung was almost restricted to 15 min post intravenous injection. The formation of emboli in the sense of passive accumulation of clusters due to very small vessel diameters [58, 59] and step by step disappearance following blood stream seems to be very unlikely due to missing relating histopathological alterations like infarctions. Whereas a very similar observation was made by Al-Jamal et al. in a magnetic tumor targeting model where high amounts of PEGylated nanocapsules (comparable size to MNPSNPs, different composition; 1 h after i.v. injection) disappeared to a large extent during further 3 h [43], Mojica Pisciotti et al. obtained higher values of PEGylated magnetite particles (comparable size, no silica shell; i.v.) in lung than in liver still after 24 h. The reason for the much longer presence in the lung in their study is probably related to the fact that the externally applied permanent magnet was not far away, placed on the tumor site (flank) during the 24 h-time period [60].

In contrast to the lung, accumulation of MNPSNPs in the liver and in the red pulp of the spleen can be attributed to a passive particle accumulation due to higher permeability of sinusoidal capillaries (100–1000 nm pore size [61, 62]) additional to active phagocytosis of macrophages [63]. Estevanato et al. showed that already one hour after intravenous administration Kupffer cells were actively involved in capture of dextran functionalized magnetite nanoparticles (approx. 10 nm in diameter) enclosing them in phagolysosomes [64]. After a few months, Perls reaction in the area of these dextran nanoparticle clusters demonstrated Fe(III) release which would pass over to the physiological iron metabolism [64]. The significant decrease around the 42nd day in the present study probably also indicates beginning degradation of MNPSNPs.

Excretion of MNPSNPs, which is assumed mainly by urine and negligibly by faeces [43], might be the reason for the fine distribution in the tubules (epithelium and lumen) of kidney at later time points, which could be visualized by MPM. Histological examination only showed MNPSNP clusters in small quantities in the glomeruli of the kidneys and tubule-associated in group 0. Natarajan et al. observed higher amounts of 100 nm radioimmunonanoparticles in kidneys than in spleens after 48 h [65].

Detected MNPSNP clusters in the brain might be sporadically located inside larger blood vessels [66, 67]. It is assumed that they did not cross the blood brain barrier and were transported via blood flow to other organs being trapped there [68–70]. In addition, MNPSNPs were not supposed to cause any damage in the brain [71–73]. Clusters in heart muscles were most likely located intravascularly.

In conclusion, intravenous administration of RITC-labeled MNPSNPs (420 µg per animal) does not seem to affect mouse physiology although temporary accumulation in different organs occurs. The combination of silica shell, PEG and size led to similar distribution as published for other nanoparticles [43, 44, 60, 63–65]. The PEGylation in order to prolong the half-life period in the blood and to avoid the observed capture in inner organs by the MPS [74] was therefore not adequate enough [75, 76]. This reduces distinctly the availability of nanoparticles in the blood stream and therewith hinders their accumulation at the desired location.

This might be one reason why the third hypothesis that a significant higher number of MNPSNPs can be accumulated at ferromagnetic implant surfaces in a magnetic field gradient could not be confirmed. In in vitro experiments from Janßen et al. (tube system filled with circulating MNPSNP suspension), it was assumed that the accumulated MNPSNP mass in the test area in passes without ferromagnetic material was caused by the exclusive power of the electromagnetic field. A slight mass increase was observed due to the use of ferromagnetic plate inducing a magnetic gradient [53] which was also described as a key factor for augmenting magnetic force [77]. The in vivo setup was expected to offer an enhanced opportunity to assess the magnetic influence of the ferritic material. The first reason for this assumption is the difference between tube and blood vessel diameter and related flow velocities. While 12.2 mm/s were used in the in vitro trial [53], much lower flow velocities exist in vivo in small animals (~ 1 mm/s [78–80]) and even in the human capillaries (< 1 mm/s [81–84]). In general, it is stated that the lower the flow velocity, the higher the amount of accumulated nanoparticles [26, 50, 85, 86]. The second reason is the higher frequency of circulating MNPSNPs passing the implantation area. In theory, with a cardiac output of approx. 15 mL/min [87, 88] an average total blood volume of 1.8 mL [89] (inclusive intravenous injection volume) from treated mice will be pumped around over 80 times in 10 min. In the above mentioned in vitro setup from Janßen et al., where MNPSNPs were trapped by similar implants and magnetic field forces in a circulating tube system, only one twentieth of the value was reached [53]. This means a much higher probability for MNPSNPs in the blood to be trapped by magnetic force in vivo. Of course, this simplified calculation is not directly transferable to the in vivo situation, which is influenced by numerous factors, but shows that aspects other than physical had probably reduced the accumulation.

The ferritic steel implant only shows a tendency of increased accumulation compared to the titanium alloy, which is far from clinical need. A nanoparticle distribution is presumed which is predominantly passive and not actively supported by the implant. Probably the vessels were temporarily leaky due to surgical insertion of implants and MNPSNPs were led by blood (unspecific, heterogeneous distribution) and the exclusive power of electromagnetic field and its gradient, which was carried out at both implant materials. The latter could also explain MNPSNP clusters between the muscle fibers in both hindlimbs. The decline after one day can be explained by the removal of MNPSNPs by the mononuclear phagocyte system (MPS). Compared to scores described by Janßen et al. where MNPSNPs were detected on the implant surface in considerable quantities even 7 days after subcutaneous injection around the implants [53], the value 7 days after intravenous administration is negligible. This significant difference is less due to phagocytosis by the MPS, but rather due to low quantities at the beginning after intravenous application, shown by higher but overall low score values in group 0. However, what was caused by the immune system are the inflammatory reactions and formation of fibrosis which were found around the implants. It is a characteristic response towards the implant as foreign body [90] and was also shown in the earlier examinations by Janßen et al. after subcutaneous administration of MNPSNPs [53]. According to biocompatibility, ferritic steel implants as a not approved material for in vivo application, seem to be promising for future designing and investigations due to missing significant difference towards the titanium alloy.

Another possible reason for the inadequate targeting result is an insufficient vascular permeability [91]. In healthy muscle tissue the continuous capillary pore size is about 6 nm [61, 92, 93]. It is unlikely that MNPSNPs of our size could widen these pores [94, 95] by means of pressure due to magnetic force and pass through by paracellular way. Qiu et al. observed that 33 nm PEG-coated nanocrystals were endocytosed by endothelial cells, which led to intracellular magnetic force while an external magnetic field was applied. This force caused a disruption of adherens junctions and consequently increased endothelial permeability [54]. If this scenario had occurred in the here presented study and inter-endothelial clefts were wide enough, residual nanoparticles in the blood would have been able to extravasate. Although MNPSNPs are negatively charged [96, 97] and hydrophilic [98] due to PEG-coating, pinocytosis (< 500 nm size [99]) could occur, albeit more slowly. In addition, caveolae-mediated endocytosis which exists in muscles, among others [100], could have functioned as transcytosis pathway [101, 102]. However, in the clinical scenario of implant infection, increased endothelial permeability is present [103, 104] and therewith overcoming the first barrier, the transfer from blood vessel into the infected tissue surrounding the implant, should probably occur.

In stent models with a magnetic source inside the vessel as well as in investigations towards particle behavior in a vessel with close externally applied magnetic field (simulation or in vitro, respectively) the following proportionalities were observed: The higher the magnetic field strength and the gradient, the particle size and concentration and the lower the fluid flow velocity and the distance between vessel and external magnet, the higher the capture efficiency of the magnet [50, 85, 86, 105–108]. In the here presented study, magnetic field application time of 10 min might have been too short [54] or the produced magnetic field strength was insufficient [77, 109–111] although many in vitro and in vivo studies used lower magnetic field strength than 1.7 T for successful targeting [43, 60, 85]. Regarding the permeability of the ferritic steel, the implant was possibly inadequate to enhance the magnetic field strength of the electromagnet and to build up a stronger gradient in the way needed. The relatively small geometry of the implant could likely drastically lower the usually higher permeability. In the case that the electromagnetic field is the dominating magnetic force, it is conceivable that MNPSNPs align themselves in this field [112] instead of being attracted by a point source and leave the region of interest after field removal. Compensating, the distance between the used ferritic implant and a blood vessel in muscle tissue or skin is about a few micrometers or less, so very small [105]. Furthermore, diameter and magnetophoretic force of nanoparticles enormously influence the accumulation [113, 114] because particles have to oppose many forces like blood flow velocity (see above), gravitation, among others [77, 110, 115]. Particle interactions with other particles or collision with blood cells and the type of protein corona might also affect the administered MNPSNPs [77, 96, 110, 115, 116]. On the contrary, no significant influence on capture efficiency was expected from the thickness of silica- and PEG-coatings of MNPSNPs according to computer simulations investigated by Lunnoo and Puangmali [113].

Although clinically relevant accumulation of MNPSNPs at the implant surface could not be shown in the present study, this concept constitutes a great potential because several factors are different in the scenario of an infected implant in humans. When implant-associated infection occurs, the vascular permeability of surrounding tissue is automatically enhanced [104] and nanoparticles should be able to accumulate in the implant region. Accordingly, the significant difference towards the control implant will arise from MNPSNPs overcoming the distance between blood vessel and implant surface only in case of occurring magnetic field gradient. Further research work, however, is needed to prevent nanoparticle clearance by the MPS. Therefore, apart from PEG, additional functionalization or coatings are necessary, e.g. binding of CD47 to the surface of the nanoparticles [76, 117, 118]. Another focus is the enhancement of magnetic properties by equipping nanoparticle cores with higher iron content [43] and design larger implants out of a highly permeable, remanent ferromagnetic material [26], probably with additional surface coatings [52]. With the manifold changes it must be considered, that superparamagnetism and a suitable size of nanoparticles are continuously guaranteed.

## Conclusion

Altogether, the intravenous application of fluorescent MNPSNPs in mice was well biocompatible, showing no clinical or significant pathomorphological alterations of inner organs up to 42 days after administration. In parallel, significant targeting of MNPSNPs from the blood to a subcutaneous magnetized ferritic steel 1.4521 implant by an externally applied magnetic field (electromagnet) was not achieved. This was especially attributed to high capture of MNPSNPs by MPS in lung, liver and spleen. Other factors contributing to the lack of MNPSNP accumulation at the implantation site might be the insufficient permeability of blood vessels in the target region and probably the implant dimensions and therewith magnetic properties in this in vivo model.

Although the goal of sufficient accumulation could not be reached, particular challenges concerning, e.g., improvement of particle properties for better biodistribution or magnetic implant properties for higher accumulation at the surface could be carved out for further studies. In this way, the presented study lays a valuable basis for the local treatment of orthopedic implant-associated infections after systemically administered nanoparticles by ID-MDT in the future.

## Data Availability

The datasets used and/or analyzed during the current study are available from the corresponding author on reasonable request

## Abbreviations

n: number
Ti90Al6V4: titanium–aluminium–vanadium alloy
MNPSNPs: magnetic nanoporous silica nanoparticles
PEG: polyethylene glycol
ID-MDT: implant-directed magnetic drug targeting
etc.: *et cetera*
MPS: mononuclear phagocyte system
et al.: *et alii*
BW: body weight
i.p.: intraperitoneal
Fe_3_O_4_: magnetite
RITC: rhodamine B isothiocyanate
*Lnn*.: *lymphonodi*–Lymph nodes
H.E.: hematoxylin–eosin
A. dest.: destilled water
p: probability
MPM: multiphoton microscopy
Fig.: figure
i.v.: intravenous
Fe: iron
e.g.: *exempli gratia*
CD47: cluster of differentiation 47
